# Depletion of enteric bacteria diminishes leukocyte infiltration following doxorubicin-induced small intestinal damage in mice

**DOI:** 10.1371/journal.pone.0173429

**Published:** 2017-03-03

**Authors:** Jacquelyn S. Carr, Stephanie King, Christopher M. Dekaney

**Affiliations:** 1 Department of Surgery, The University of North Carolina at Chapel Hill, Chapel Hill, North Carolina, United States of America; 2 Department of Molecular Biomedical Sciences, NC State University, Raleigh, North Carolina, United States of America; National Cancer Institute, UNITED STATES

## Abstract

**Background & aims:**

While enteric bacteria have been shown to play a critical role in other forms of intestinal damage, their role in mediating the response to the chemotherapeutic drug Doxorubicin (Doxo) is unclear. In this study, we used a mouse model of intestinal bacterial depletion to evaluate the role enteric bacteria play in mediating Doxo-induced small intestinal damage and, more specifically, in mediating chemokine expression and leukocyte infiltration following Doxo treatment. An understanding of this pathway may allow for development of intervention strategies to reduce chemotherapy-induced small intestinal damage.

**Methods:**

Mice were treated with (Abx) or without (NoAbx) oral antibiotics in drinking water for four weeks and then with Doxo. Jejunal tissues were collected at various time points following Doxo treatment and stained and analyzed for apoptosis, crypt damage and restitution, and macrophage and neutrophil number. In addition, RNA expression of inflammatory markers (TNFα, IL1-β, IL-10) and cytokines (CCL2, CC7, KC) was assessed by qRT-PCR.

**Results:**

In NoAbx mice Doxo-induced damage was associated with rapid induction of apoptosis in jejunal crypt epithelium and an increase weight loss and crypt loss. In addition, we observed an increase in immune-modulating chemokines CCL2, CCL7 and KC and infiltration of macrophages and neutrophils. In contrast, while still positive for induction of apoptosis following Doxo treatment, Abx mice showed neither the overall weight loss nor crypt loss seen in NoAbx mice nor the increased chemokine expression and leukocyte infiltration.

**Conclusion:**

Enteric bacteria play a critical role in Doxo-induced small intestinal damage and are associated with an increase in immune-modulating chemokines and cells. Manipulation of enteric bacteria or the damage pathway may allow for prevention or treatment of chemotherapy-induced small intestinal damage.

## Introduction

Doxorubicin (Doxo) is a highly morbid chemotherapeutic drug utilized as first-line treatment for several types of cancer, including subtypes of breast cancer, soft tissue sarcomas, and lymphomas.[1–3] Its major mechanism of action is DNA intercalation, which prevents DNA replication, ultimately causing DNA damage and cell cycle arrest. One of the major side effects of the drug is mucositis, deep ulceration of the mucosal lining of the digestive tract. This side effect can be dose-limiting and can sometimes render patients unable to complete their chemotherapeutic regimens. Medications like ondansetron, an anti-emetic, can somewhat assuage the symptoms of mucositis, but there are no effective treatments for mucositis barring discontinuation of chemotherapy. For this reason, research into successful approaches for reduction of the development of mucositis is needed.

We and others have previously demonstrated in mice that Doxo induces a significant, rapid increase of apoptosis in small intestinal crypt epithelium.[4–6] This increase in apoptosis is accompanied by an increase in permeability of the intestinal epithelia barrier[7] followed by significant mucosal damage, characterized by crypt loss and villus blunting, and a subsequent repair phase during which crypts lengthen and hypertrophy. About one week after treatment, normal morphology within the jejunum is restored. Our recent work has further demonstrated the importance of enteric bacteria in this process as germ free (GF) mice do not appear to demonstrate the characteristic sequelae of damage following Doxo, suggesting that mucositis is mitigated in the absence of bacteria.[8]

Increasing evidence demonstrates that the microbiota contribute to other causes of small intestinal damage, like inflammatory bowel diseases,[9–12] NSAID-associated intestinal damage,[13–15] and ischemia reperfusion injury.[16–18] Interestingly, some studies suggest that the presence of enteric bacteria protects from the development damage, while others suggest that the presence of enteric bacteria is detrimental and contributes to inflammation and damage. Others have explored the murine response to Doxo in models of limited bacterial signaling. Nigro et al. treated mice with muramyl-dipeptide, a Nod2 agonist and peptidoglycan common to all bacteria, and concluded that epithelial restitution following Doxo is Nod2 dependent.[19] In contrast, Kaczmarek et al. observed less small intestinal damage in TLR2 and TLR9 knockout mice following Doxo, concluding that bacterial signaling via these receptors was necessary for damage.[20] Furthermore, their study demonstrated that TLR2 or TLR9 deficiency abrogated the accumulation of CD45^+^ cells following Doxo treatment suggesting a correlation between enteric bacteria, Doxo treatment, and infiltration of leukocytes.

In this study, we tested the hypothesis that depletion of enteric bacteria in mice would result in decreased infiltration of leukocytes into the intestinal lamina propria following Doxo treatment. To do this we utilized a mouse model of intestinal bacterial depletion which involved a regimen of high-dose oral antibiotics to more closely mirror a clinically plausible human model and to evaluate the relationship between enteric bacteria and lamina propria leukocytes within the context of Doxo-induced damage. The aims of the study were to corroborate our previously published work that bacteria are required for induction of Doxo-induced small intestinal damage as assessed by weight loss, crypt loss, and crypt hyperplasia[8], to demonstrate that induction of an immune-modulating cascade involving increased expression of the chemokines CCL2 and CCL7 correlates with increased infiltration of macrophages and neutrophils, and to determine whether this response is enteric bacteria dependent. The results of this study may provide important progress towards understanding the mechanism(s) by which enteric bacteria mediate Doxo-induced mucositis, and suggest multiple means by which enteric bacterial signaling can be manipulated in order to reduce chemotherapy-induced mucositis.

## Material and methods

### Animals

Adult male and female C57BL/6 mice were purchased from Jackson Laboratories (Bar Harbor, ME) and used between 8–12 weeks of age. An age-matched cohort of female C57BL/6 mice were raised under germ free (GF) conditions in the National Gnotobiotic Research Center at the University of North Carolina at Chapel Hill, and used between 8–12 weeks of age. A cohort of male C57BL/6 mice (Abx) were treated with oral antibiotics as described by Rakhoff-Nahoum et al.: ampicillin (APP Pharmaceuticals, Lake Zurich, IL) at 1 mg/mL, neomycin (Medisca, Irving, TX) at 1 mg/mL, metronidazole (Fluka, St Louis, MS) at 1 mg/mL, and vancomycin (Hospira, Lake Forest, IL) at 500 μg/mL dissolved in iodinated water supplemented with 15% strawberry syrup for four weeks.[9] An age-matched cohort of male C57BL/6 mice (NoAbx) received iodinated water with 10% syrup alone. The differences in percentage of syrup between Abx and NoAbx were due to an adjustment based on the amount of water consumed by each group. To evaluate ingestion of water, bottle volumes were weighed twice weekly, and to evaluate weight changes in Abx and NoAbx mice, each mouse was weighed twice weekly. Mice from both NoAbx and Abx cohorts ingested equivalent water and gained equivalent weight. To ensure depletion of enteric bacteria in Abx mice as compared to NoAbx mice, feces were collected from each mouse in each cage at weeks 3 and 4, and cultured on LB media in aerobic and anaerobic conditions (S1 Fig). Cages with detectable colonies were eliminated from all experiments. Experimental procedures were approved by the Institutional Animal Care and Use Committees of The University of North Carolina at Chapel Hill and NC State University.

### Doxo treatment and tissue processing

Mice were given a single intraperitoneal (IP) injection of Doxo (Pharmacia & Upjohn Co., Kalamazoo, MI) at a dose of 20 mg/kg body weight, which we have previously reported induces reproducible sequela of jejunal damage in mice.[21] Animals were sacrificed 0, 6 hours, 24 hours, 3 days or 5 days after Doxo treatment and weighed at the time of sacrifice. The jejunum was isolated and flushed with 5 mL ice-cold Hank’s balanced salt solution (Gibco, Waltham, MA). Portions of jejunum were fixed in 10% buffered formalin and embedded in paraffin for histologic analyses. Additional portions of jejunum were snap frozen in liquid nitrogen for RNA and protein extraction.

### Histology

Formalin-fixed paraffin embedded specimens were oriented to provide sections perpendicular to the long axis of the bowel, and three 5μm sections were used for evaluating general morphology. Crypts were selected for scoring crypt loss and crypt depth on the basis that a single, continuous layer of epithelium followed from crypt base to villus base. Crypt loss was scored by counting the total number of crypts present in the complete cross-section of tissue, as well as the total number of crypts present in a representative 1000 um length. Crypt depth was calculated by measuring the depth of 10–20 crypts per cross-section, with a total of three cross-sections per animal, using Axio Imager software on images captured using an Axio Imager A1 microscope and an AxioCam MRC 5 high resolution camera (Carl Zeiss Microimaging, Inc. Thornwood, NY) at 64X. Apoptosis was scored by H&E staining based on the presence of pyknotic bodies within 10–20 crypts per cross-section, with a total of three cross-sections per animal, and confirmed by immunoflourescence staining for active caspase 3.[22]

### Immunostaining

For immunohistochemistry, slides were deparaffinized, rehydrated, and incubated in 3% hydrogen peroxide for 15 min at room temperature (RT) to quench endogenous peroxidase activity. Sections were treated to heat-induce epitope retrieval (Antigen Unmasking Solution cat. # H-3300, Vector Laboratories, Burlingame, CA) and allowed to cool to RT. To stain actively proliferating cells, primary antibody (rabbit anti-phospho histone H3 cat. 9701 Cell Signaling Technology, Danvers, MA, USA) was applied to each section at a 1:300 dilution and incubated overnight at 4°C. Sections were then washed and incubated with biotinylated goat anti-rabbit secondary antibody for 30 min at RT. For macrophage staining, primary antibody (rat anti-F4/80 cat. 6640 Abcam, Cambridge, MA) was applied to each section at a 1:200 dilution and incubated for 90 min at 60°C. Sections were then washed and incubated with biotinylated goat anti-rat secondary antibody for 60 min at RT. For neutrophil staining, primary antibody (rat anti-neutrophil cat. 2557 Abcam, Cambridge, MA) was applied to each section at a 1:200 dilution and incubated for 90 min at 60°C. Sections were then washed and incubated with biotinylated goat anti-rat secondary antibody for 60 min at RT. After secondary antibody was removed, all slides were washed and incubated in Vectastain ABC Elite reagent (Vector Laboratories, Burlingame, CA) for 30 min and then developed in a DAB substrate solution. Data are expressed as number of positive cells per crypt. For immunofluorescence, slides were deparaffinized, rehydrated, treated to antigen retrieval in 10% NGS/TRIS for 30 min, and allowed to come to RT. Sections were washed and incubated with rabbit anti-cleaved caspase 3 (cat. no. 9661, Cell Signaling Technology, Danvers, MA) at a 1:800 dilution overnight at 4°C. Sections were then washed and incubated with goat anti-rabbit Cy3 secondary antibody for 2 hours at RT. Finally, sections were mounted using Vectashield Mounting Medium with DAPI (H-1200 Vector Laboratories, Burlingame, CA) and evaluated using an Axio Imager A1 microscope and an AxioCam MRC 5 high resolution camera.

### RNA isolation and quantitative RT-PCR

Total RNA was isolated using Trizol (Invitrogen, Carlsbad, CA) following manufacturer instructions. cDNA was generated utilizing Applied Biosystems High Capacity Reverse Transcription cDNA kit (Applied Biosystems, Waltham, MA), and quantitative real-time PCR was performed in duplicate using Applied Biosystems StepOne Plus real time PCR system and TaqMan Universal PCR Master Mix (Applied Biosystems, Foster City, CA). Primer and probe sets for TNFα (Mm00443258_m1), IL1β (Mm00434228_m1), and IL10 (Mm01288386_m1), CCL2 (Mm00441242_m1), CCL7 (Mm00443113_m1), KC (Mm04207460_m1) and β-actin (Mm00607939_m1) were purchased from Applied Biosystems. Data were analyzed using the ΔΔC_t_ method with normalization to β-actin mRNA as the constitutive mRNA. Mean β-actin mRNA did not differ significantly between mice. Averaged mRNA from four untreated adult C57BL/6J mice was used as the reference standard.

### Statistics

All quantitative results are presented as means ± SE. All data were subjected to two-way ANOVA with correction for multiple comparisons using the Fisher’s procedure. For all histologic scoring, 30–60 crypts were counted per mouse and averaged to yield a single mean, and means for each mouse in the group were averaged to yield a single mean per group. For all comparisons, a *P* value of < 0.05 was considered significant. All authors had access to the study data and reviewed and approved the final manuscript.

## Results

### Depletion of enteric bacteria does not alter doxorubicin-induced apoptosis, but ameliorates downstream intestinal damage

**Fig 1:**
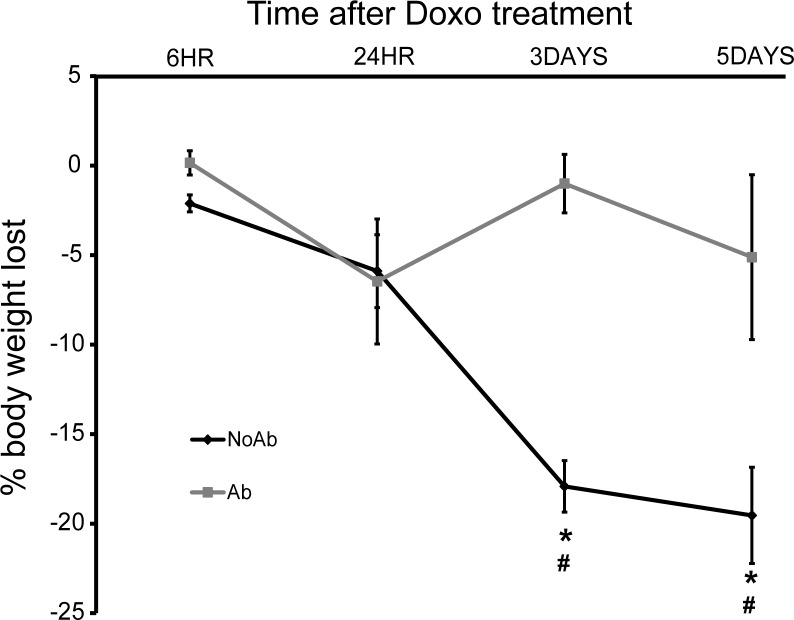
Doxorubicin (Doxo) induces weight loss in non-antibiotic treated (NoAbx) mice, but does not induce weight loss in antibiotic-treated (Abx) mice. NoAbx and Abx were treated with a single dose of intraperitoneal Doxo at 20 mg/kg. Percent body weight loss at 6 hours, 24 hours, 3 days and 5 days was calculated from weight at injection. n = 3–5 per time point per group. * = values significantly different from respective control; P<0.05

To determine the effect of doxorubicin on animals depleted of enteric bacteria, mice treated with high-dose oral antibiotics (ampicillin, neomycin, vancomycin, metronidazole) or vehicle were given a single IP dose of Doxo and sacrificed at multiple time points following treatment. The first parameter we evaluated was weight loss, as we have previously shown weight loss to be a reliable marker of Doxo-induced pathology.[23] Consistent with our previous findings, NoAbx mice demonstrated significant, time-dependent weight loss following Doxo. Conversely, Abx mice did not experience significant weight loss at any time point following Doxo (Fig 1).

**Fig 2:**
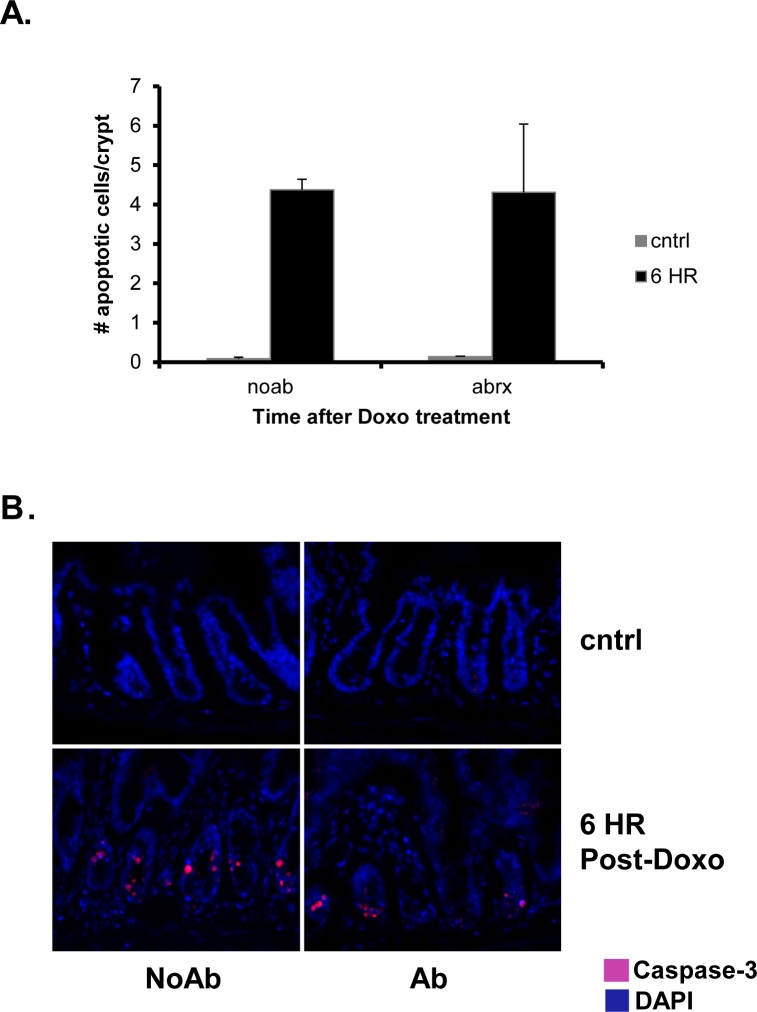
Doxorubicin (Doxo) induces equivalent apoptosis in antibiotic-treated (Abx) and non-antibiotic treated (NoAbx) mice. A. Quantification of the number of apoptotic cells per crypt in Abx and NoAbx jejunal tissue from control mice and 6 hours after Doxo treatment. n = 3–4 per time point per group. * = values significantly different from respective control; P<0.05. B. Immunofluorescence staining at 64X demonstrating active caspase 3-positive cells (pink) in Ab and NoAbx jejunal tissue from control mice and 6 hours after Doxo treatment.

We next assessed apoptosis 6 h following administration of Doxo, as our previous work demonstrated that the peak of apoptosis occurs at this time point.[21] Interestingly, we found an equivalent number of apoptotic cells within the jejunal crypts of both NoAbx and Abx mice. This was demonstrated by quantification of the number of apoptotic bodies per crypt on H&E staining (Fig 2A) as well as active caspase-3 immunofluorescence (Fig 2B). These findings suggest that Doxo was able to exert its immediate, anti-neoplastic function of cell death regardless of the presence of enteric bacteria.

**Fig 3:**
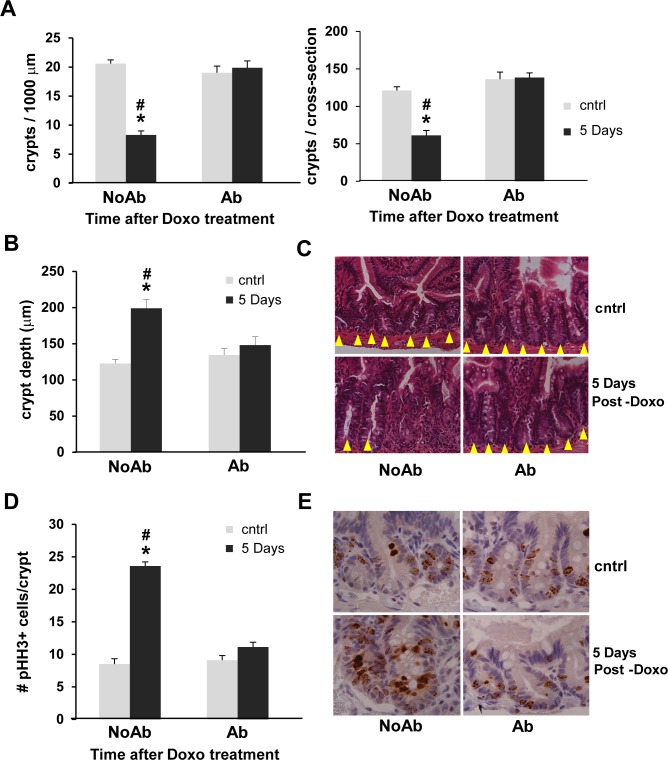
Doxorubicin (Doxo) induces crypt loss followed by crypt deepening and proliferation in non-antibiotic treated (NoAbx) mice, but not in antibiotic-treated (Abx) mice. A. Number of crypts was quantified in control mice and 5 days following treatment with Doxo. Results expressed as total number of crypts per 1000 μm (left) and total number of crypts per cross-section (right). B. Crypt depth (μm) was quantified in control mice and 5 days following treatment with Doxo. C. H&E staining of representative cross-sections at 64X. Yellow arrowheads denote individual crypts. D. Proliferation was evaluated by quantification of pHH3+ cells in control mice and 5 days following treatment with Doxo. E. IHC staining of representative cross-sections at 64X. Brown cells denote pHH3+ cells. n = 3–5 per time point per group. * = values significantly different from respective control; P<0.05. # = values significantly different between groups (NoAbx & Abx) within a specific time point; P<0.05.

Characterization of the injury and restitution response within jejunal crypts following Doxo revealed a significant disparity between NoAbx and Abx mice. Five days following treatment with Doxo, NoAbx mice demonstrated significant loss of crypts, while Abx mice did not. This was determined by quantifying the number of total crypts per jejunal cross-section, as well as the number of crypts in a fixed length of cross-section (1000 μm), in order to account for potential differences in the circumference of the jejunum between mice (Fig 3A). An increase in crypt depth, which serves as a marker of crypt restitution following Doxo, was evident in NoAbx mice, but Abx mice did not demonstrate this increase (Fig 3B). Representative H&E micrographs of crypt number and depth are shown in Fig 3C. Likewise, proliferation, another marker of crypt restitution, was observed in NoAbx, but not Abx, mice by quantification of phosphohistone H3 positive cells per crypt (Fig 3D). Representative IHC-stained micrographs are depicted in Fig 3E.

### Doxo does not induce a robust inflammatory response in NoAbx mice, but does induce an increase in immune-modulating cytokines that is blunted in Abx mice

**Fig 4:**
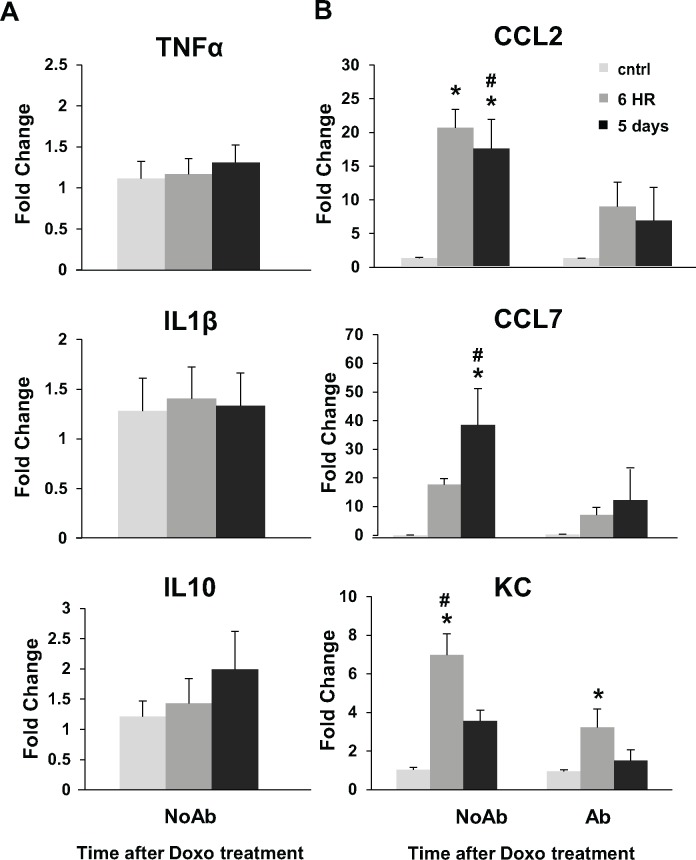
Doxorubicin (Doxo) does not induce an increase in inflammatory cytokines but does induce an increase in immune-modulating chemokines in non-antibiotic treated (NoAbx) mice; this increase is dampened in antibiotic-treated (Abx) mice. A. Fold change in mRNA of TNFα, IL1β, and IL10 in NoAbx jejunal tissue from control mice, 6 hours and 5 days after Doxo treatment. n = 5–8 mice per time point. B. Fold change in mRNA of CCL2, CCL7, and KC in Abx and NoAbx jejunal tissue from control mice, 6 hours and 5 days after Doxo treatment. n = 3–5 mice per time point per group. * = values significantly different from respective control; P<0.05. # = values significantly different between groups (NoAbx & Abx) within a specific time point; P<0.05.

Because inflammation has been shown to play a significant role in other models of intestinal damage like NSAID abuse [13, 14], ischemia/reperfusion [16–18] and irradiation [9, 24], we next evaluated whether inflammation plays a role in Doxo-induced injury. As demonstrated in Fig 4A, mRNA expression of key pro- and anti-inflammatory markers TNFα, IL1β, and IL10 did not change in the jejunal tissue of NoAbx mice at several time points after the administration of Doxo. Conversely, there was a significant fold-increase in the mRNA expression of immune-modulating chemokines CCL2, CCL7, and KC (mouse homolog of CXCL1) following Doxo in NoAbx mice (Fig 4B). This increase was significantly blunted in Abx mice (Fig 4B).

### Doxo induces an increase in immune-modulating cells in NoAbx, but not Abx, mice

**Fig 5:**
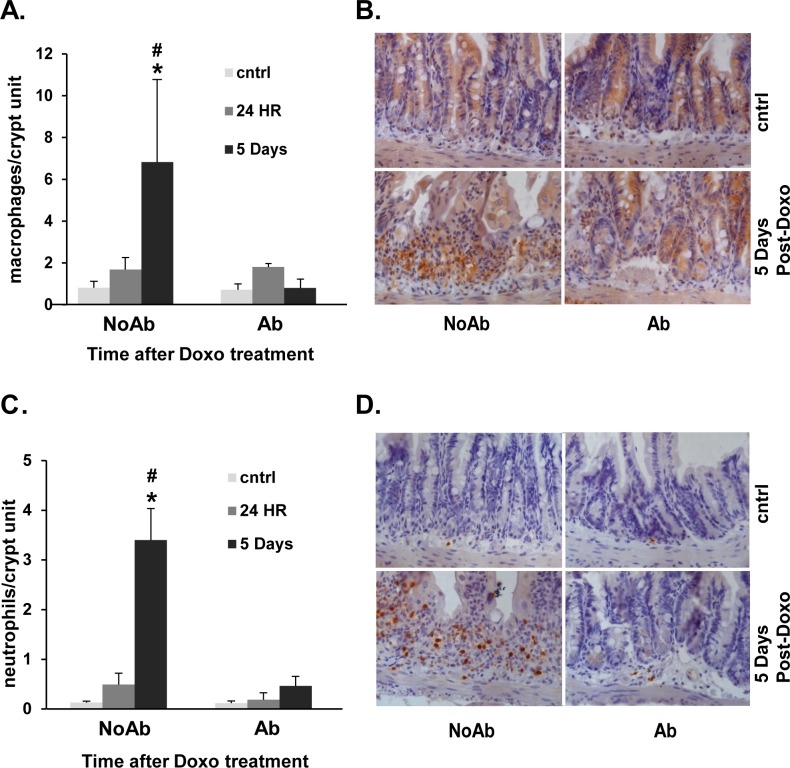
Doxorubicin (Doxo) induces an influx of macrophages and neutrophils in non-antibiotic treated (NoAbx) mice, but not in antibiotic-treated (Abx) mice. A. Quantification of the number of macrophages (F4/80+) per crypt unit in Abx and NoAbx jejunal tissue from control mice, 24 hours and 5 days after Doxo treatment. B. IHC staining of representative cross-sections with brown cells denoting F4/80+ cells. C. Quantification of the number of neutrophils (anti-neut+) per crypt unit in Abx and NoAbx jejunal tissue from control mice, 24 hours and 5 days after Doxo treatment. D. IHC staining of representative cross-sections with brown cells denoting anti-neut+ cells. n = 3–4 per time point per group. * = values significantly different from respective control; P<0.05. # = values significantly different between groups (NoAbx & Abx) within a specific time point; P<0.05.

Since CCL2, CCL7 and KC have been shown to recruit neutrophils and macrophages to sites of tissue damage [13], we next assessed whether this increase in immune-modulating chemokines was accompanied by an increase in immune cells. Indeed, we found that both macrophages and neutrophils were increased in the submucosa surrounding jejunal crypts of NoAbx, but not Abx, mice following Doxo. This was determined by quantification of macrophage anti-F4/80 positivity (Fig 5A) and neutrophil anti-neutrophil positivity (Fig 5C) within the crypt units of jejunal tissue. Representative micrographs are depicted in Fig 5B and 5D.

**Fig 6:**
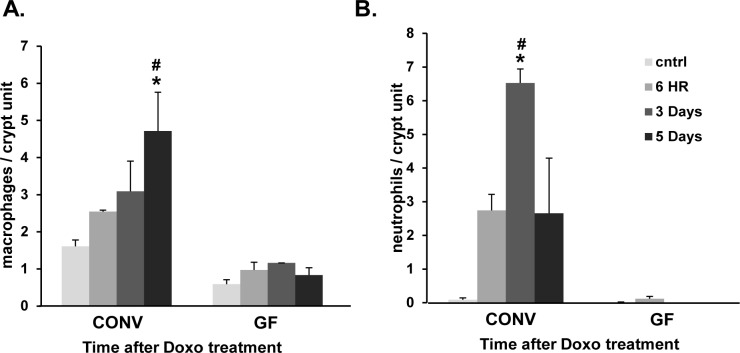
Doxorubicin (Doxo) induces an influx of macrophages and neutrophils in conventionally-raised (CONV) mice, but not in germ free (GF) mice. A. Quantification of the number of macrophages (F4/80+) per crypt unit in CONV and GF jejunal tissue from control mice, 6 hours, 3 days and 5 days after Doxo treatment. B. Quantification of the number of neutrophils (anti-neut+) per crypt unit in CONV and GF jejunal tissue from control mice, 6 hours, 3 days and 5 days after Doxo treatment. n = 3 per time point per group. * = values significantly different from respective control; P<0.05. # = values significantly different between groups (CONV & GF) within a specific time point; P<0.05.

To address the potential contribution of the antibiotic treatment (verses the depletion of enteric bacteria) on these results, this experiment was repeated in conventionally-raised (CONV) and germ free (GF) mice, and findings were equivalent (Fig 6). Thus, our results suggest that the protective effect on antibiotic treatment observed is due to depletion of bacteria.

## Discussion

The role of enteric bacteria in mediating small intestinal damage due to a number of conventional clinical sources, like irradiation, NSAIDs and chemotherapy, has yet to be fully defined. In this study we depleted enteric bacteria to further delineate the role enteric bacteria play in damage response associated with Doxo treatment. We report that, similar to what we have previously reported in germ free mice,[8] enteric bacteria are required for doxorubicin-induced small intestinal damage, and that the elaboration of immune-modulating chemokines and cells associated with this damage also relies upon the presence of enteric bacteria.

To perform these studies, we employed a previously described method to deplete the enteric bacteria of mice with high dose oral antibiotics.[9] Critics of the germ-free model argue that germ-free animals do not isolate the effect of eliminating microbiota because bacteria are required for the development of a normal immune response.[25, 26] Since germ free animals are born in a germ free environment, their immune responses are altered at baseline, and therefore the effects of a lack of bacteria cannot be separated from the effects of an atypical immune response. Critics of the antibiotic model argue that the effects of the antibiotics themselves cannot be separated from the effects of depleting enteric bacteria, though this criticism has been well-refuted.[27] Additionally, in the present study, our findings in germ free mice parallel our findings in antibiotic-treated mice, suggesting our data indeed represent the effect of depletion of enteric bacteria.

Though our data suggest that enteric bacteria are required for the development of Doxo-induced mucositis, enteric bacteria do not appear to be required to see the immediate, apoptotic effects of Doxo. Both NoAbx and Abx mice show equivalent apoptosis 6 hrs after Doxo administration, a time point at which we have previously shown peak apoptosis following Doxo.[4, 28] Apoptosis has been utilized as a standard marker of cytotoxicity for multiple chemotherapeutic drugs, including doxorubicin, as well as for irradiation.[5, 29, 30] Persistent apoptosis in Abx mice thus suggests that Doxo is able to exert its immediate, anti-tumor properties despite the absence of bacteria, a fact that may be critical when considering means of manipulating the enteric bacteria in order to decrease chemotherapy-induced mucositis.

Because multiple models of murine small intestinal damage suggest a crucial role for the inflammatory response, we evaluated the role of inflammation in Doxo-induced damage. Intestinal damage secondary to murine DSS-induced colitis[9, 10, 31], ischemia/reperfusion injury[16, 17] total body irradiation[24] and infectious colitis[32] are all associated with a robust inflammatory response, and specifically, with robust expression of the inflammatory cytokines, IL1β, IL10, and TNFα. Surprisingly, there was no increase in these cytokines at any time-point after Doxo treatment in our study. This was in contrast to the other intestinal damage models noted above and to the increase in TNFα and IL1β seen in mouse mononuclear cells[33] and serum[34] with the well-studied chemotherapeutic irinotecan. Nonetheless, in the present study, the absence of these factors in the small intestine following Doxo suggests that inflammation may not significantly contribute to Doxo-induced mucositis.

In contrast to our findings on inflammatory markers, our study suggests that local production of immune-modulating chemokines CCL2, CCL7 and KC, coupled with the increase in macrophages and neutrophils within the lamina propria, plays a key role in Doxo-induced mucositis. CCL2, CCL7 and KC are secreted by monocytes, macrophages and dendritic cells and recruit monocytes, T-cells, macrophages and neutrophils to sites of injury. Macrophages have been shown to be critical in the pathogenesis of murine intestinal graft vs host disease[35] and DSS colitis[36], while neutrophils appear to be critical for the development of ischemia/reperfusion injury in mice[37] as well as humans.[38] Conversely, a distinct population of macrophages have been shown to play a protective role in small intestinal damage due to murine DSS colitis[31] and infectious schistosomiasis.[39] These discrepancies may be explained by the existence of two distinct populations of macrophages: classically activated M1 macrophages that are stimulated by IFNγ and act via Th1 cells to generate an inflammatory response, and alternatively activated M2 macrophages that are stimulated by IL17 and act via Th2 cells and play a role in tissue repair and remodeling.[31, 40] The precise role of macrophages in Doxo-induced mucositis remains unclear, as the macrophages present in our model may be *contributing* to the damage response, or they may be *responding* to damage and participating in the repair response. In either case, our data indicate that macrophage recruitment to sites of injury is a key component of the response to Doxo-induced damage, and that enteric bacteria constitute the trigger for this response.

Based on our current findings, we believe the likely mechanism for Doxo-induced small intestinal damage involves penetration of the epithelial barrier by bacteria or bacterial products with subsequent generation of chemokines via bacterial product interaction with non-epithelial cells of the lamina propria. This mechanism requires disruption of the small intestinal epithelial barrier, leading to increased permeability, following Doxo. Studies have demonstrated an association between epithelial apoptosis and increased permeability of the intestinal barrier in rats following Doxo[7] and irinotecan[41], and in epithelial HT-29 monolayers following treatment with the chemotherapeutic Camptothecin.[42] Increased epithelial permeability has been implicated in other forms of small intestinal injury, like ischemia/reperfusion[17] and inflammatory bowel disease[11] in mice, and NSAID-associated damage in humans.[15, 43] As discussed above, the present study confirms that Doxo induces apoptosis regardless of the presence of bacteria. We thus hypothesize that Doxo induces an apoptosis-associated increase in permeability of the epithelial barrier, followed by translocation of bacterial products to the subepithelial tissue, where resident cells initiate a cascade of CCL2, CCL7 and KC production that ultimately leads to infiltration of macrophages and neutrophils. Accordingly, without luminal bacteria, the apoptosis-associated increase in permeability does not launch this response; there are no bacterial products present to penetrate the epithelia.

One alternative to eliminating enteric bacteria to ameliorate chemotherapy-induced mucositis is to augment the bacterial census with probiotics. For example, Wang et al. investigated the efficacy of *Streptococcus thermophiles* TH-4 against Doxo-induced mucostis in rats and found that while pretreatment with the probiotic reduced weight loss seven days following Doxo treatment compared with rats treated with Doxo alone it had not impact on overall severity score.[44] In contrast, Whitford et al. demonstrated that TH-4 significantly decreased the severity score of 5-FU induced intestinal damage in rats suggesting that probiotic efficacy may be associated with the chemotherapeutic agent.[45] Similarly, an investigation by Yeung et al. demonstrated effectiveness of probiotics such as *Lactobacillus casei* variety *rhamnosus* or *Lactobacillus acidophilus* and *Bifidobacterium bifidum* at reducing 5-FU-induced mucositis in mice.[46] Studies such as these and others point to the intestinal microbiota as not only an important component of the pathogenesis of chemotherapy-induced mucositis but also as a potential means to ameliorate it.[47] Furthermore, they highlight that much more study is needed of both chemotherapy-induced pathogenesis and the roles enteric bacteria play in small intestinal damage.

There are no known preventative strategies or treatments for chemotherapy-induced mucositis. The data reported in this study suggest that enteric bacteria/bacterial metabolites in conjunction with infiltrating macrophages and neutrophils play critical roles in the mechanism of Doxo-induced intestinal damage. Elucidating the pathway(s) of microbial dependence for small intestinal damage following Doxo could provide opportunities for clinical manipulation of the enteric microbiota or constituents of the pathway which would lead to reduced damage. Reduction of Doxo-associated damage could, in turn, impact cancer treatment strategies by allowing increased dosing potentially increasing the efficacy of clinical treatment.

## Supporting information

S1 Fig Treatment with high dose oral antibiotics depletes enteric bacteria. A. C57BL6 mice from 8–12 weeks of age were treated with oral ampicillin, vancomycin, neomycin and metronidazole, or sham, for 30 days. Equivalence between sham and treatment groups was validated by weighing bottles twice per week (suggesting equal ingestion) and weighing of mice twice per week (suggesting lack of systemic effects of antibiotics). Feces were cultured twice weekly in aerobic and anaerobic conditions to evaluate bacterial growth. B. Feces from cages of mice that received antibiotics (AB) and mice that received sham water (NOAB) were pooled and plated in aerobic and anaerobic conditions. AB cages demonstrated less growth than NOAB cages in aerobic (shown) and anaerobic (not shown) conditions at week 3 (shown) and week 4 (not shown) of treatment.
(EPS)
